# 427. Bloodstream infections among adult COVID-19 patients in Colombian hospitals between 2020-2021

**DOI:** 10.1093/ofid/ofad500.497

**Published:** 2023-11-27

**Authors:** Christian Pallares, María Virginia Villegas, Jessica Porras, Karen Melissa Ordoñez, Elsa Yasmín Vente, Erika Vanessa Patiño, Sandra Milena Gómez

**Affiliations:** Centro Médico Imbanaco de Cali, Cali, Valle del Cauca, Colombia; Universidad El Bosque, Cali, Valle del Cauca, Colombia; Molecular Genetics and Antimicrobial Resistance Unit – International Center of Microbial Genomics, Universidad El Bosque, Bogota, Distrito Capital de Bogota, Colombia; ESE Hospital Universitario San Jorge de Pereira, Pereira, Risaralda, Colombia; Clínica Imbanaco grupo quirónsalud, Cali, Valle del Cauca, Colombia; Clínica la Estancia, Popayán, Cauca, Colombia; Clínica San Francisco, Tuluá, Valle del Cauca, Colombia

## Abstract

**Background:**

Coronavirus is one of the major pathogens that primarily targets the human respiratory system. In COVID-19 patient’s, bloodstream infections (BSI) have not been extensively studied in Latin-America. Given the impact of COVID-19 pandemic in Colombia and the high rate of hospitalizations, information about risk factors and ways to prevent BSI among COVID-19 patients is urgently needed. This investigation aimed to identify risk factors in BSI in hospitalized patients with COVID-19.

**Methods:**

A case-control design was used; cases were patients with a PCR confirmed COVID-19 infection and BSI. Controls were patients with a PCR confirmed COVID-19 who did not have a BSI during their hospitalization. Information about demographic, clinical characteristics, comorbidities and exposures that could be related to BSI were reviewed. Descriptive statistics were calculated, odds ratios (ORs) and 95% confidence intervals were estimated using multivariable conditional logistic regression models. In accordance with Colombian regulations -Resolution 8430 of the Ministry of Health of 1993- this project was classified as risk-free.

**Results:**

306 patients (153 cases and 153 controls) from four participating hospitals were included. Cases and controls had similar average age (62-63 years), median length of stay (19-25 days), comorbidities and similar Newscore scale regarding severity on admission to the hospital; all cases and 89% of controls required at least one invasive device. Cases were more likely to have required an orotracheal tube (97% vs. 81%; p< 0.05), central venous catheter (95% vs. 82%; p< 0.05), or urinary catheter (98% vs. 80%; p< 0.05) compared with controls. Cases had higher previous antimicrobials use (91% vs. 77%; p< 0.05), pronation (93% vs. 81%; p< 0.05) and chlorhexidine bath (58% vs. 83%, p< 0.05) than controls. However,no statistically significant differences in mortality between cases and controls was found. In the multivariate analysis, urinary catheter used (OR= 11.05; IC95% 1.18–103.18) was a risk factor and chlorhexidine bath (OR=0.47; IC95% 0.25–0.88) was a protective factor for BSI.

CASE AND CONTROL CHARACTERISTICS

**Figure:**
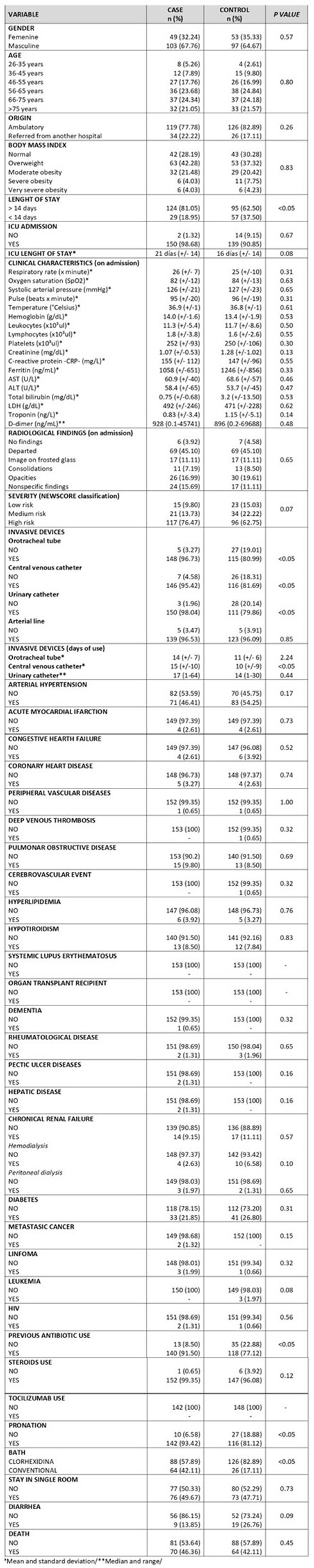

BIVARIATE AND MULTIVARIATE ANALYSIS

**Figure:**
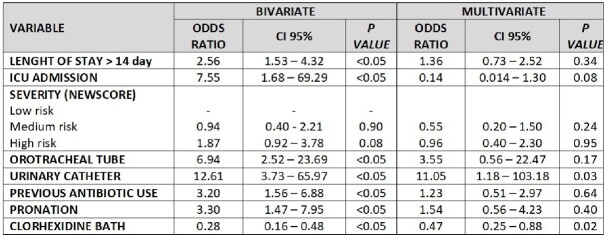

**Conclusion:**

COVID-19 patients are more propense to BSI, but bathing with chlorhexidine and care and monitoring of invasive devices are modifiable factors to minimize the risk of infections.

**Disclosures:**

**Christian Pallares, MD, MSc**, 3M: Advisor/Consultant|3M: Honoraria|MSD: Advisor/Consultant|MSD: Grant/Research Support|MSD: Honoraria|Pfizer: Advisor/Consultant|Pfizer: Grant/Research Support|Pfizer: Honoraria|Westquímica: Advisor/Consultant|Westquímica: Grant/Research Support|Westquímica: Honoraria **María Virginia Villegas, n/a**, MSD: Advisor/Consultant|MSD: Grant/Research Support|MSD: Honoraria|Pfizer: Advisor/Consultant|Pfizer: Grant/Research Support|Pfizer: Honoraria|Westquímica: Advisor/Consultant|Westquímica: Grant/Research Support|Westquímica: Honoraria **Karen Melissa Ordoñez, n/a**, Pfizer: Advisor/Consultant|Pfizer: Honoraria

